# Ternary Phenolate-Based Thiosemicarbazone Complexes of Copper(II): Magnetostructural Properties, Spectroscopic Features and Marked Selective Antiproliferative Activity against Cancer Cells

**DOI:** 10.3390/molecules29020431

**Published:** 2024-01-16

**Authors:** Iman K. Al-Salmi, Musa S. Shongwe

**Affiliations:** Department of Chemistry, College of Science, Sultan Qaboos University, P.O. Box 36, Al-Khod 123, Muscat, Oman

**Keywords:** mononuclear and dinuclear ternary copper(II) complexes, thiosemicarbazone, X-ray structures, tetragonal distortion, intermolecular forces, spectroscopy, selective potent cytotoxicity

## Abstract

The new diprotic ligand 3,5-di-*tert*-butylsalicylaldehyde 4-ethyl-3-thiosemicarbazone, abbreviated H_2_(3,5-*t*-Bu_2_)-sal4eT, exists as the thio-keto tautomer and adopts the *E*-configuration with respect to the imine double bond, as evidenced by single-crystal X-ray analysis and corroborated by spectroscopic characterisation. Upon treatment with Cu(OAc)_2_·H_2_O in the presence of either 2,9-dimethyl-1,10-phenanthroline (2,9-Me_2_-phen) or 1,10-phenanthroline (phen) as a co-ligand in MeOH, this thiosemicarbazone undergoes conformational transformation (relative donor-atom orientations: *syn,anti* → *syn,syn*) concomitantly with tautomerisation and double deprotonation to afford the ternary copper(II) complexes [Cu{(3,5-*t*-Bu_2_)-sal4eT}(2,9-Me_2_-phen)] (**1**) and [Cu_2_{3,5-*t*-Bu_2_)-sal4eT}_2_(phen)] (**2**). Crystallographic elucidation has revealed that complex **1** is a centrosymmetric dimer of mononuclear copper(II) complex molecules brought about by intermolecular H-bonding. The coordination geometry at the copper(II) centre is best described as distorted square pyramidal in accordance with the trigonality index (τ = 0.14). The co-ligand adopts an axial–equatorial coordination mode; hence, there is a disparity between its two Cu–N coordinate bonds arising from weakening of the apical one as a consequence of the tetragonal distortion. The axial X-band ESR spectrum of complex **1** is consistent with retention of this structure in solution. Complex **2** is a centrosymmetric dimer of dinuclear copper(II) complex molecules exhibiting intermolecular H-bonding and π-π-stacking interactions. The two copper(II) centres, which are 4.8067(18) Å apart and bridged by the thio-enolate nitrogen of the quadridentate thiosemicarbazonate ligand, display two different coordination geometries, one distorted square planar (τ_4_ = 0.082) and the other distorted square pyramidal (τ_5_ = 0.33). Such dinuclear copper(II) thiosemicarbazone complexes, which are crystallographically characterised, are extremely rare. In vitro, complexes **1** and **2** outperform cisplatin as antiproliferative agents in terms of potency and selectivity towards HeLa and MCF-7 cancer cell lines.

## 1. Introduction

Thiosemicarbazones are multi-purpose hydrazone ligands of considerable interest in coordination chemistry [1]. Their characteristic functionality feature R^1^R^2^C=N–N(H)–C(=S)–NR^3^R^4^ is derived from the straightforward single-step Schiff-base condensation reaction between an aldehyde or a ketone and a thiosemicarbazide. Amongst their fascinating structural attributes is their coordination versatility, arising from their propensity to undergo concomitant base-/metal-assisted tautomeric transformation and deprotonation as a means to meet charge-neutrality requirements. Moreover, a given thiosemicarbazone is capable of exhibiting different denticities and coordination modes as demanded by the metal centre [2,3,4,5,6,7,8,9]. By strategically employing aldehydes or ketones with moieties bearing donor atoms of interest in appropriate coordination positions, they can be tailor-designed for specially desired hetero-donor environments, coordination spheres [10], physicochemical features and biological properties [3,8,9,11,12,13,14,15,16,17,18,19,20,21,22,23,24,25,26,27]. Substituent groups can impart electronic effects to thiosemicarbazone complexes with interesting impacts on physicochemical and pharmacological properties. The literature is replete with examples of thiosemicarbazone complexes exhibiting a broad spectrum of pharmacological properties including antitumour [3,8,9,11,12,13,14,15,16,17,18,19,20,21,22], antibacterial [20,21,23], antiviral [24,25], antifungal [23,26] and antimalarial properties [22,27]. 

Thiosemicarbazones stabilise predominantly metal ions from the *p-*, *d*- and *f*-blocks [1]; some of the transition-metal thiosemicarbazone complexes exhibit fascinating magneto-structural [28] and catalytic [29] properties amongst other features. Electrochemically, for copper (*Z* = 29; [Ar]4*s*^1^3d^10^), which is the subject of this paper, Schiff-base complexes shuttle between +1 and +2 oxidation states (Cu^II^/Cu^I^ redox couple) [8,15,18,20,30]. Thiosemicarbazone complexes whose cyclic voltammograms exhibit these redox couples with potentials lying within the biologically accessible redox potential window ranging from −0.4 to +0.8 V vs. NHE [30] are of pharmacological importance in that they have the ability to generate intracellular reactive oxygen species (ROS) [8,15,18,20,30] desirable for apoptotic cytotoxicity. Ternary mononuclear complexes of copper(II) of the type [CuL(N,N-donor-L)]^n+^ abound, where L represents a neutral or anionic polydentate primary ligand and N,N-donor-L stands for a heterocyclic bidentate N,N-donor chelating co-ligand such as 1,10-phenanthroline (phen), 2,2′-bipyridine (bipy), dipyridoquinoxaline (dpq), dipyridophenazine (dppz) or derivatives of these [31,32,33,34,35,36,37,38,39,40,41,42,43,44,45,46,47,48,49,50,51,52,53,54,55,56,57,58]. Such coordination compounds are of considerable interest partly because of their potential to exhibit DNA binding/cleavage [34,39,41,42,43,44,50,51,53,54,55], anticancer [39,43,44,49,53,54] and antimicrobial [35,36,37,40] activities. Some have been explored as models for metallo-enzymes [33,45] while others have been designed to investigate structural and spectroscopic features of interest [4,32,38,46,47,48,52,56,57,58]. There is a paucity of crystallographically characterised ternary copper(II) *thiosemicarbazone* complexes of this type [4,31,32,33,34,35,36,37,38,39]. 

In this work, we have synthesised and structurally characterised the ligand 3,5-di-*tert*-butylsalicylaldehyde 4-ethyl-3-thiosemicarbazone, H_2_(3,5-*t*-Bu_2_)-sal4eT. Reaction of copper(II) ion with this ligand and the co-ligand 2,9-dimethyl-1,10-phenanthroline (2,9-Me_2_-phen) or 1,10-phenanthroline (phen) in equimolar amounts produced the ternary copper(II) complex [Cu{(3,5-*t*-Bu_2_)-sal4eT}(2,9-Me_2_-phen)] (**1**) or [Cu_2_{3,5-*t*-Bu_2_)-sal4eT}_2_(phen)] (**2**), respectively. The 3D structures have been determined through single-crystal X-ray analyses. Whereas complex **1** is mononuclear, complex **2** is dinuclear. In the crystal lattice, each exists as a dimer arising from two symmetrically related intermolecular H-bonding interactions. To the best of our knowledge, [Cu_2_{3,5-*t*-Bu_2_)-sal4eT}_2_(phen)] (**2**) is one of only two examples of crystallographically characterised dinuclear copper(II) thiosemicarbazone complexes of this kind. The other is [Cu_2_(sal4eT)_2_(bipy)], previously designated [Cu_2_(L^2^)_2_(bipy)] (bipy = 2,2′-bipyridine) [4]. While complex **2** is a centrosymmetric dimer of dinuclear complexes, the crystallographic asymmetric unit of [Cu_2_(sal4eT)_2_(bipy)] consists of two independent intramolecularly hydrogen-bonded dinuclear complex molecules. In both **2** and [Cu_2_(sal4eT)_2_(bipy)], the two copper(II) ions are in different coordination spheres. These two complexes differ in the five-coordinate geometry. The antiproliferative activity of complexes **1** and **2** along with H_2_(3,5-*t*-Bu_2_)-sal4eT has been tested against the cancer cell lines MCF-7 (human breast adenocarcinoma) and HeLa (human cervical carcinoma). Whereas the ligand is inactive against HeLa and MCF-7 cancer cells, the complexes are highly potent and selective.

## 2. Results and Discussion

### 2.1. Synthesis and Chemical Identification of the Thiosemicarbazone Ligand

**Scheme 1:**
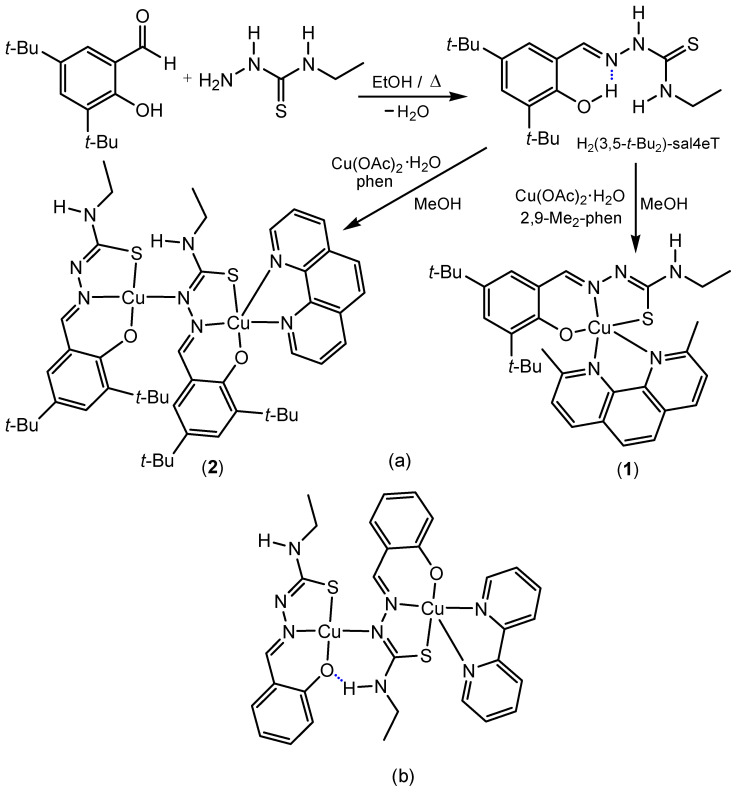
(**a**) Illustration of synthetic routes to H_2_(3,5-*t*-Bu_2_)-sal4eT and complexes **1** and **2**; (**b**) comparative structural representation of [Cu_2_(sal4eT)_2_(bipy)] [4].

The thiosemicarbazone H_2_(3,5-*t*-Bu_2_)-sal4eT was synthesised from equimolar amounts of 3,5-di-*tert*-butylsalicylaldehyde and 4-ethyl-3-thiosemicarbazide through the usual single-step Schiff-base condensation reaction in refluxing absolute ethanol. The resultant light yellow solution afforded long lustrous colourless needles upon slow evaporation of the solvent under ambient conditions over a span of several days. Unlike the synthesis of pyridyl-based thiosemicarbazones [8,13,14,15], this phenolic thiosemicarbazone was produced straightforwardly in high yield without requiring acid-catalysis (Scheme 1). The chemical identity of this ligand was ascertained through microanalysis along with electrospray ionisation (ESI) mass spectrometry. The ESI mass spectrum exhibits a parent peak at *m*/*z* = 334.3 [M − H^+^]^−^ in the negative-ion mode (Figure S1) and at *m*/*z* = 336.3 [M + H^+^]^+^ in the positive-ion mode, consistent with the molecular mass (M = 335.51 amu). 

### 2.2. FT-IR and NMR Spectroscopic Characterisation of the Thiosemicarbazone Ligand 

**Figure 1:**
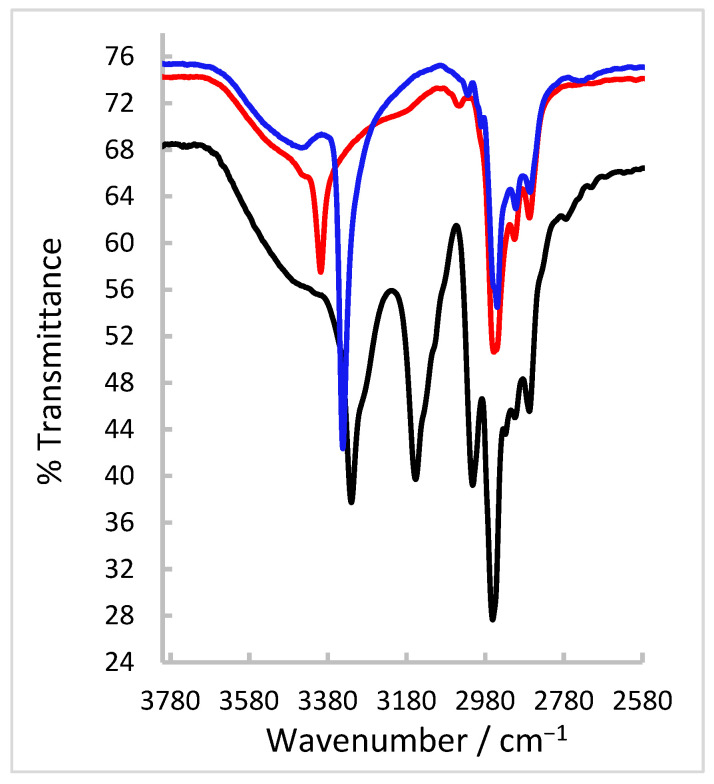
FT-IR spectra of H_2_(3,5-*t-*Bu_2_)-sal4eT (black line), [Cu{3,5-*t*-Bu_2_)-sal4eT}(2,9-Me_2_-phen)] (**1**) (blue line) and [Cu_2_{3,5-*t*-Bu_2_)-sal4eT}_2_(phen)] (**2**) (red line) in the region 3780–2580 cm^−1^.

That H_2_(3,5-*t-*Bu_2_)-sal4eT exists as the thio-keto (thione) tautomer in the solid state is demonstrated by the prominent IR absorption band at 3157 cm^−1^, indicative of the thio-amide ν(N–H) (Figure 1 and Figure S2a). The associated thio-carbonyl bond is characterised by the vibrational band with a stretching frequency of 1032 cm^−1^. Indeed, the absence of a vibrational band at around 2600 cm^−1^ due to ν(S–H) [10] excludes the possibility of the occurrence of the thio-enol tautomer. At 3320 cm^−1^ in the IR spectrum, an absorption band occurs that is ascribable to the N–H stretching of the terminal secondary amino group of the thiosemicarbazone. A characteristic feature of Schiff bases is the imine bond whose presence in H_2_(3,5-*t-*Bu_2_)-sal4eT is evidenced by the absorption at 1609 cm^−1^ typifying ν(C=N). The *tert*-buyl C–H stretches are conspicuous given their characteristic pattern of absorption bands in the range of 2867–2960 cm^−1^ [59]. Contributing to the intensity of these absorptions are the C–H vibrations of the *N*-ethyl substituent group. The sharp absorption band with a stretching frequency of 3013 cm^−1^ is attributable to the aromatic ν(C–H). Finally, the broad band at around 3500 cm^−1^ is typical of phenolic O–H vibrations. 

The ^1^H-NMR spectrum of H_2_(3,5-*t-*Bu_2_)-sal4eT was recorded in DMSO-*d*_6_ (Figure S3a) at a radiofrequency of 700 MHz with TMS as an internal reference standard (δ = 0). The broad peak at δ 9.98 assignable to the hydrazinic proton reveals that the thione tautomer of this thiosemicarbazone remains intact in solution. The phenolic proton, which is represented by the singlet at δ 11.27, is the most deshielded on account of its intramolecular interaction with the imine nitrogen atom. The aldimine proton is associated with the sharp singlet at δ 8.28. A broad resonance shaped like an unresolved triplet occurs at δ 8.48 and is attributable to the proton of the amino group between the thio-carbonyl and ethyl groups. The aromatic protons in positions 4 and 6 resonate as doublets at δ 7.13 and 7.29, respectively, with identical coupling constants (*J* = 2.38 Hz). The *N*-ethyl group is characterised by partially overlapping quartet signals at δ 3.59 (*J* = 6.55 Hz) and a triplet resonance at δ 1.16 (*J* = 7.14 Hz) corresponding to the methylene protons (in non-equivalent environments) and the methyl protons, respectively. Finally, the protons of the 3-*tert*-butyl and 5-*tert*-butyl substituent groups have singlet signals with the chemical shifts at δ 1.41 and 1.27, respectively.

Interestingly, when the ^1^H-NMR spectrum of H_2_(3,5-*t-*Bu_2_)-sal4eT is measured in deuterated methanol (CD_3_OD), the resonances of the –OH and the two –NH protons disappear (Figure S3b), indicative of rapid exchange of each of these for deuterium on the NMR timescale. The chemical shift at δ 8.14 of the imine –C*H*=N singlet is the most downfield in this spectrum. The aromatic protons in positions 4 and 6 appear as doublets (δ 7.14, *J* = 2.38 Hz and δ 7.38, *J* = 2.44 Hz, respectively). The ethyl –N(H)–C*H*_2_C*H*_3_ protons are observed as quartet (δ 3.70, *J* = 7.18 Hz) and triplet (δ 1.24, *J* = 7.19 Hz) signals, respectively, whereas the 3-*tert*-butyl and 5-*tert*-butyl protons occur as singlet peaks at δ 1.44 and 1.30, respectively.

The ^13^C-NMR spectrum of H_2_(3,5-*t*-Bu_2_)-sal4eT, recorded in DMSO-*d*_6_ at 176 MHz (Figure S4), exhibits a resonance for the thio-carbonyl carbon at δ 176.29, confirming the existence of this thiosemicarbazone as the thione tautomer in this solution. At δ 147.54, a signal occurs representing the imine carbon atom. All the aromatic carbon atoms are accounted for in the range of δ 117.75–153.11, with the phenolic carbon being the most deshielded. The signals of the *N*-ethyl methylene and methyl carbon atoms appear at δ 38.73 and 14.59, respectively. Finally, the *tert*-butyl carbon atoms have resonances in the range of δ 29.44–34.71. 

### 2.3. Single-Crystal X-ray Structural Determination of the Thiosemicarbazone Ligand

**Figure 2:**
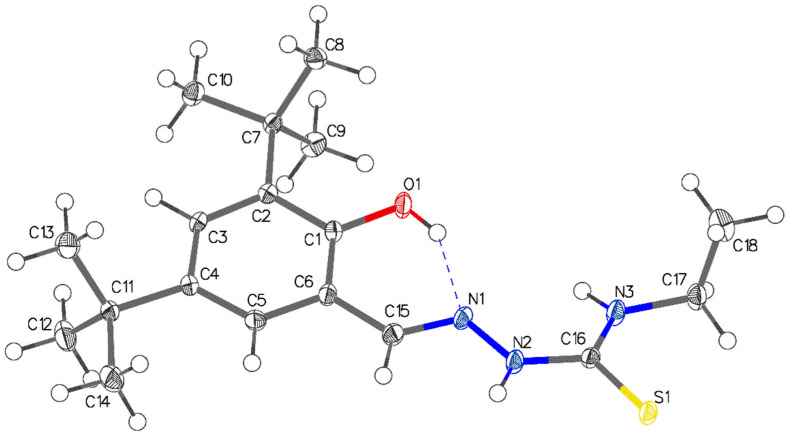
X-ray crystal structure of H_2_(3,5-*t*-Bu_2_)-sal4eT.

Definitive evidence for the solid-state 3D structure of the ligand was obtained through single-crystal X-ray analysis. A colourless needle amenable to X-ray diffraction was grown from a solution of H_2_(3,5-*t*-Bu_2_)-sal4eT in EtOH at room temperature. X-ray data collection was performed at 100 K. Crystal data, details of data collection and parameters for structural solution and refinement are compiled in Table 1. Evidently, this thiosemicarbazone ligand crystallised in the monoclinic space group *P*2_1_/*c* with four molecules in the unit cell. The X-ray crystal structure is depicted in Figure 2 while selected bond distances and angles are presented in Table 2. The distance of the Schiff-base bond C=N [C(15)–N(1) = 1.2904(19) Å] lies within the range observed for normal imine bonds [1.26–1.30 Å] [13,14,15,19,20,60,61,62,63,64,65,66,67,68,69] in non-coordinated ligands. Upon reduction in the Schiff base, the imine double bond becomes a single bond (C–N) with a distance of ~1.47 Å [65]. The C(16)–S(1) distance of 1.7029(14) Å verifies the occurrence of the thione tautomer. Literature values for the distance of the thio-carbonyl bond in free thiosemicarbazone ligands range typically from 1.65 to 1.70 Å [13,14,15,19,20,60,61,62,63,64,65,66,67,68,69] (even longer if involved in bifurcated H-bonding) [67]. Both imine nitrogen and thio-carbonyl carbon are sp^2^-hybridised and the angles around them reflect the angular (with a lone pair) and trigonal planar geometries about these two atoms. The hydrazinic N–N bond [N(1)–N(2) = 1.3854(16) Å] is somewhat longer than most of those reported for other non-coordinated thiosemicarbazones and is consistent with single-bond character.

One of the prominent structural features of interest is the intramolecular H-bonding interaction between the phenolic –O–H group and the imine nitrogen atom [O(1)–H(1)···N(1):O1–H1 = 0.84 Å, H1···N1 = 1.98 Å, O1···N1 = 2.7221(15) Å, O1–H1···N1 = 147.4°]. Indeed, the vast majority of Schiff bases derived from 2-hydroxybenzaldehydes, 2-hydroxyacetophenones, 2-hydroxybenzophenones, 2-hydroxypropiophenones, etc., exhibit this intramolecular electrostatic force. It is well-established that pyridyl-/phenol-based thiosemicarbazones can adopt an *E*- or *Z*-configuration with respect to the imine double bond. Moreover, they can also orient themselves in different conformations as a consequence of free rotation about the C(py/phenol)–C(imine) (i.e., C(6)–C(15) in this structure) single bond and the amide N(H)–C(=S) (i.e., N(2)–C(16) in this structure) single bond. Thus the potential donor atoms can be positioned *anti* or *syn* relative to each other. Examples of crystallographically observed orientations of phenolic thiosemicarbazones, *viz. E(syn,anti)* [60,62,63], *E(syn,syn)* [61,64] and *E(ant,anti)* [63], are shown in Figure S5. The structure of H_2_(3,5-*t*-Bu_2_)-sal4eT is consistent with the *E*-configuration; the phenolic –OH group and the imine nitrogen are positioned *syn* to each other while the thione sulphur points to the opposite side in an *anti-*orientation relative to the imine nitrogen. 

### 2.4. Synthesis and Chemical Identification of the Copper(II) Thiosemicarbazone Complexes

**Figure 3:**
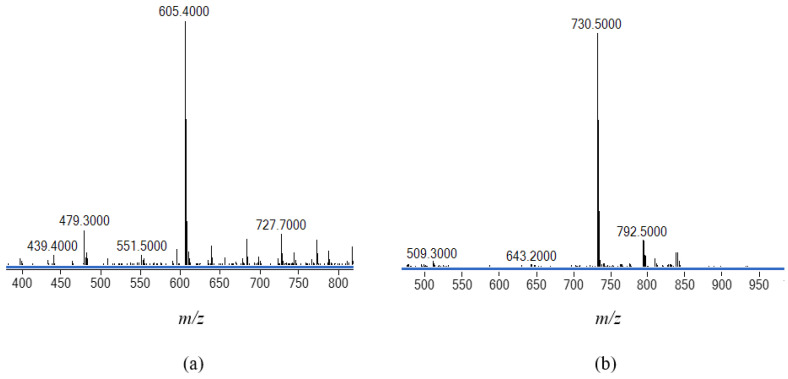
ESI mass spectra of (**a**) [Cu{(3,5-*t-*Bu_2_)-sal4eT}(2,9-Me_2_-phen)] (**1**) in the positive-ion mode and (**b**) [Cu_2_{(3,5-*t*-Bu_2_)-sal4eT}_2_(phen)] (**2**) in the negative-ion mode using MeOH as the matrix.

Reaction of H_2_(3,5-*t*-Bu_2_)-sal4eT with a molar equivalent of Cu(OAc)_2_·H_2_O in refluxing MeOH, followed immediately by addition of a stoichiometric amount of 2,9-dimethyl-1,10-phenanthroline (2,9-Me_2_-phen) or 1,10-phenathroline (phen) with brief heating of the resultant dark olive green solution, afforded the mononuclear copper(II) complex [Cu{(3,5-*t*-Bu_2_)-sal4eT}(2,9-Me_2_-phen)] (**1**) or the dinuclear copper(II) complex [Cu_2_{(3,5-*t*-Bu_2_)-sal4eT}_2_(phen)] (**2**), respectively. The chemical formulations of these two ternary complexes were established through elemental analyses. The mass spectra of the complexes were measured in MeOH. The positive-ion ESI mass spectrum of complex **1** presented in Figure 3a shows a molecular peak at *m*/*z* = 605.4 in agreement with the molecular mass of this complex (605.25 amu). As regards the dinuclear complex (**2**), the parent ion was not detected; however, the ESI spectrum revealed important structural information from the fragmentation pattern. In the negative mode, the spectrum shows a minor peak at *m*/*z* = 792.5 consistent with the loss of the phen co-ligand. At *m*/*z* = 730.5, a major peak occurs that is ascribable to the fragment [Cu{H(3,5-Bu_2_)-sal4eT}{(3,5-Bu_2_)-sal4eT}]^−^ (Figure 3b). On the other hand, the positive-ion ESI spectrum exhibits a peak at *m*/*z* = 732.6 attributable to the fragment [Cu{(3,5-*t*-Bu_2_)-sal4eT}_2_]^+^. Further dissociation affords the fragment [Cu{(3,5-*t*-Bu_2_)-sal4eT}]^+^ observed at *m*/*z* = 397.2, signifying the loss of one of the thiosemicarbazonate ligands. That complexes **1** and **2** are molecular has been demonstrated through the negligible value of the molar electrical conductivity (Λ*_M_* ~3–5 Ω^−1^ cm^2^ mol^−1^) of their nonelectrolyte solutions in MeOH, EtOH, DMF and DMSO at room temperature [70].

### 2.5. FT-IR Spectroscopy and Magnetic Susceptibility Measurements 

A comparison of the IR spectra of the thiosemicarbazone ligand and its copper(II) complexes (**1** and **2**) in Figure 1 and Figure S2 clearly shows the absence of the vibrational band of the hydrazinic N–H bond from the spectra of the complexes. The disappearance of the hydrazinic proton coupled with the shift in the stretching frequency of the absorption band of the carbon–sulphur bond from 1032 cm^−1^ for the ligand to 842 and 858 cm^−1^ for **1** and **2**, respectively, is indicative of tautomerisation and deprotonation of the thiosemicarbazone upon coordination to the copper(II) ion, as is indeed necessary for charge-neutrality of the resultant complexes. The presence of the N–H group attached to the terminal ethyl group is proven by the occurrence of sharp absorption bands at 3398 and 3342 cm^−1^ in the spectra of **1** and **2**, respectively. The wavenumbers of the imine bond for **1** and **2** complexes are somewhat lower than that of the free ligand [ν(C=N): 1598 and 1599 cm^−1^ vs. 1609 cm^−1^], consistent with coordination of the imine donor atom. Interestingly, the ν(N–N) absorptions for the ligand and complexes **1** and **2** virtually coincide (1172, 1170 and 1169 cm^−1^, respectively), implying minimal delocalisation of π-electrons, if any, along the ligand backbone in the complexes. Finally, the other ligand IR absorption patterns, especially those of the *tert*-butyl C–H bonds (2850–2960 cm^−1^), are retained.

Complexes **1** and **2** are paramagnetic with a single unpaired electron at the metal centre in the ground state. The room-temperature effective magnetic moment [µ_eff_ = (8χ_M_)^½^] of the mononuclear complex (**1**) is 1.83 µ_B_. It is comparable with the spin-only value [µ*_S_* = {4*S*(*S* + 1)}^½^, where *S* = ½] and lies within the range of literature values [45,50,54,56,57,58,59]. In contrast, for the dinuclear complex (**2**), µ_eff_ = 2.38 µ_B_ at room temperature, which is close to the spin-only value for two magnetically uncoupled d^9^ paramagnetic centres [{4*S*_1_(*S*_1_ + 1) + 4*S*_2_(*S*_2_ + 1)}^½^, where *S*_1_ = *S*_2_ = ½]. 

### 2.6. Single-Crystal X-ray Analyses of the Ternary Copper(II) Complexes

For the complexes [Cu{(3,5-*t-*Bu_2_)-sal4eT}(2,9-Me_2_-phen)] (**1**) and [Cu_2_{(3,5-*t*-Bu_2_)-sal4eT}_2_(phen)] (**2**), X-ray diffraction data were collected on a single crystal at 100 K employing Cu-Kα radiation (λ = 1.54178 Å). Crystal data together with details of data collection and structural refinement are presented in Table 1. Selected bond distances and angles are given in Table 3. Whereas complex **1** crystallised in the monoclinic space group *P*2_1_/*n* with *Z* = 4, complex **2** did so in the triclinic space group *P*$\bar{1}$ with two complex molecules in the unit cell. Both complexes **1** and **2** are free of solvent molecules of crystallisation.

**Figure 4:**
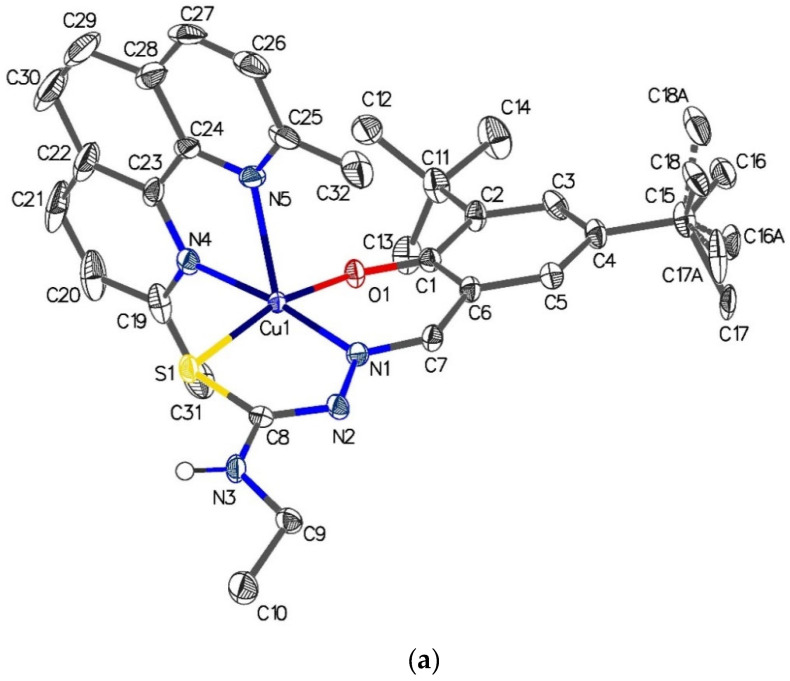

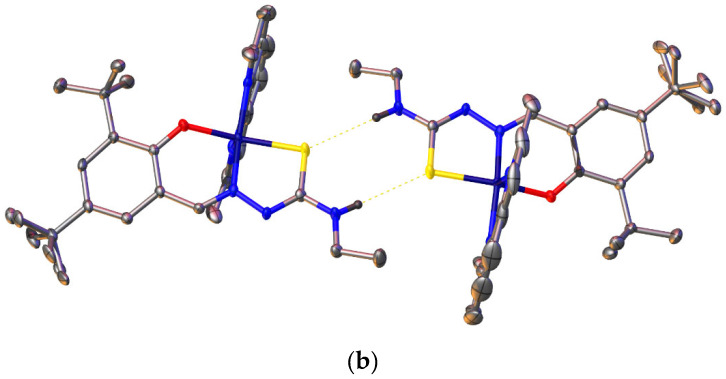
(**a**) Molecular structure of [Cu{(3,5-*t-*Bu_2_)-sal4eT}(2,9-Me_2_-phen)] (**1**) and (**b**) illustration of dimerisation of the mononuclear complex molecules.

The crystal structure of [Cu{(3,5-*t-*Bu_2_)-sal4eT}(2,9-Me_2_-phen)], depicted in Figure 4, reveals that this complex exists as a centrosymmetric dimer of mononuclear molecular ternary complexes of copper(II). The dimerisation occurs via two intermolecular hydrogen-bonding interactions involving the –N^4^–H group of one complex molecule and the thio-enolate sulphur of the other complex molecule (Figure 4b) [N(3)–H(3)···S(1): N–H = 0.88 Å, H···S = 2.64 Å, N···S = 3.482(3) Å, N–H–S = 159.9° (symmetry code: 1 − *x*, 1 − *y*, −*z*)]. The charge-neutrality of this ternary complex implies that the thiosemicarbazone been doubly deprotonated upon complexation. Indeed, transformation of the ligand from the thio-keto tautomer to the thio-enolate anion is demonstrated through the changes to the lengths of the pertinent bonds of the thio-amide. The thio-amide N–C bond [N(2)–C(16): 1.3499(18) Å in the free ligand] has shortened considerably upon complexation [N(2)–C(8): 1.313(4) Å in complex **1**] while the thio-carbonyl (C=S) bond [C(16)–S(1): 1.7029(14) Å in the ligand] has converted to the thio-enolate C–S^−^ bond in the complex [C(8)–S(1) = 1.739(3) Å]. Carbon–nitrogen bonds with double-bond character have been reported to have distances in the range 1.27–1.32 Å [66,71,72,73] when the N donor atom is coordinated to a central metal ion. On the other hand, typical lengths of carbon–sulphur bonds with single-bond character in thio-enolate complexes are in the range 1.72–1.77 Å [66,71,72,73]. The distances of the imine C=N [C(7)–N(1) = 1.296(4) Å] and the hydrazinic N–N [N(1)–N(2) = 1.400(4) Å] in **1** are normal with regard to their respective bond orders.

The five-coordinate geometry at the copper(II) centre arises from the tridentate coordination of the thiosemicarbazonate ligand with the donor atoms, namely phenolate oxygen, imine nitrogen and thio-enolate sulphur, arranged meridionally, and the bidentate coordination of the 2,9-Me_2_-phen co-ligand oriented nearly perpendicularly relative to the primary ligand. The axial–equatorial coordination mode of the pyridyl nitrogen atoms of 2,9-Me_2_-phen leads to the construction of a coordination sphere best described as distorted square pyramidal in accordance with the trigonality index [τ = (β − α)/60°] [74] of ~0.14, the largest two angles β and α being in the basal plane. The axial Cu–N bond is considerably longer than the one lying equatorially [Cu–N_ax (co-ligand)_ = 2.308(3) Å vs. Cu–N_eq (co-ligand)_ = 2.057(3) Å]. This elongation of the axial coordinate bond is attributable to the tetragonal distortion at the metal centre. Invariably, square pyramidal [31,32,33,34,35,36,37,38,39,40,41,42,43,45,46,47,48,49,50,51,52] and octahedral [44,53] ternary complexes with axial–equatorial coordination of a bidentate co-ligand (bipy, phen or their derivatives) are subject to the Jahn–Teller effect evidenced by this structural feature. In some cases, even binary bis(chelate) copper(II) complexes [75] with two potentially tridentate ligands experience this effect, causing one of the ligands to coordinate bidentately due to considerable weakening of one of the axial Cu–L bonds. The magnitude of the disparity in the Cu–N distances of the asymmetrically coordinated N,N-donor co-ligand is in the range of ~0.22–0.32 Å. Moreover, the complex cation of tris(1,10-phenanthroline)copper(II) perchlorate [76] exhibits Jahn–Teller distortion (Cu–N bond averages: Cu–N_ax_ ~2.33 Å vs. Cu–N_eq_ ~2.04 Å) whereby the axial Cu–N_phen_ bonds are elongated to the same extent as those in the above-mentioned square pyramidal copper(II) ternary complexes. In contrast, it has been crystallographically proven that the two Cu–N bonds of N,N-donor co-ligands in square pyramidal and octahedral complexes where they lie on the equatorial plane are virtually equivalent as neither is subject to the Jahn–Teller effect [54,55,56,57,58]. In addition, in the complex [Cu{N(CN)_2_}(phen)_2_]^+^ [77] with a distorted *trigonal bipyramidal* geometry at the metal centre, the phen Cu–N distances are virtually indistinguishable from each other as the Jahn–Teller effect does not apply. The copper(II) ion in [Cu{(3,5-*t-*Bu_2_)-sal4eT}(2,9-Me_2_-phen)] (**1**) is displaced out of the mean basal plane [N(1), S(1), O(1), N(4)] towards the apical phen N(5) donor atom by 0.1998(12) Å. Finally, the magnetostructural behaviour of this complex is consistent with half occupancy of the *d_x_*2_–*y*_2 orbital in the ground state. 

**Figure 5:**
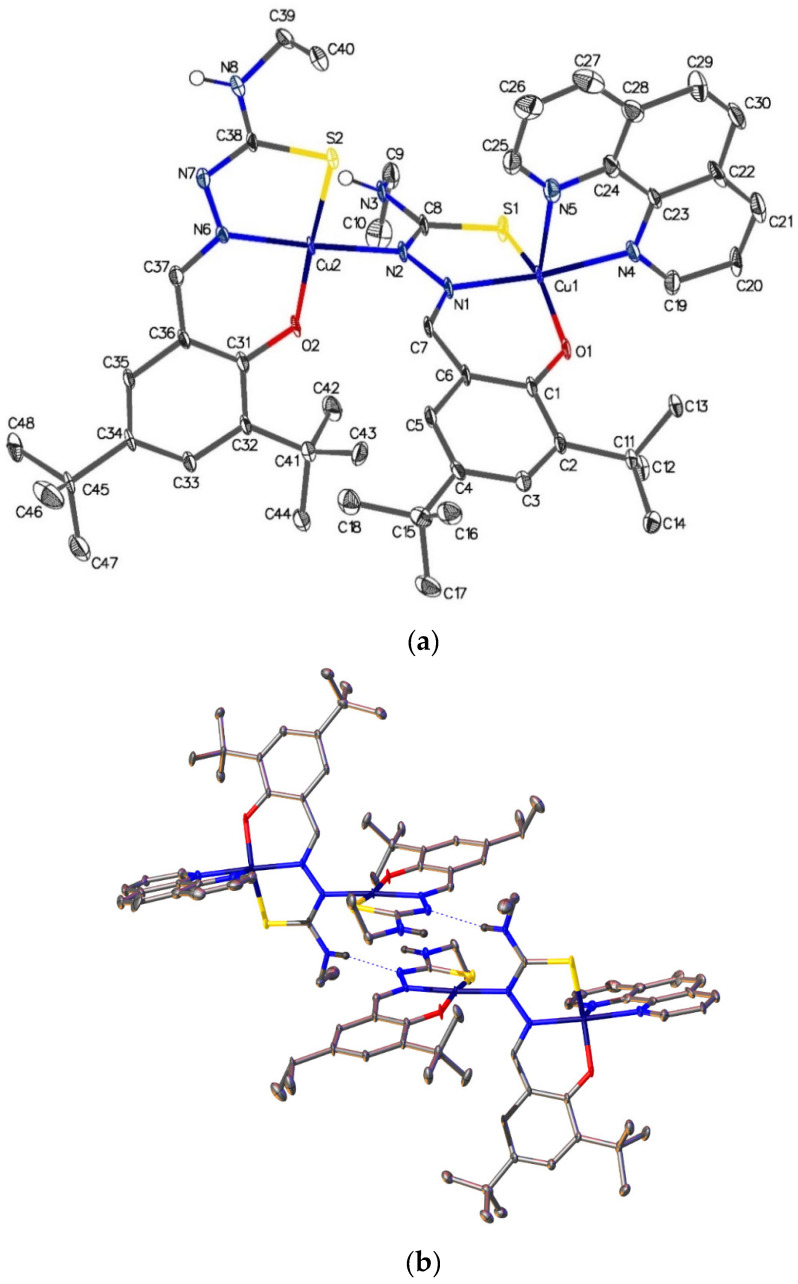
(**a**) Molecular structure of [Cu_2_{(3,5-*t-*Bu_2_)-sal4eT}_2_(phen)] (**2**) and (**b**) illustration of dimerisation of the dinuclear complex molecules.

As can be seen from Figure 5, [Cu_2_{(3,5-*t*-Bu_2_)-sal4eT}_2_(phen)] (**2**) exists in the crystal lattice as a centrosymmetric dimer of dinuclear molecular ternary complexes of copper(II) stabilised mainly by two types of intermolecular forces. The linkage of two dinuclear complex molecules occurs through two H-bonds between the N^4^–H group of one dinuclear molecule and the imine nitrogen of another dinuclear molecule [N(3)–H(3)···N(7): N–H = 0.88 Å, H···N = 2.17 Å, N···N = 2.964(11) Å, N–H···N = 150.5° (symmetry code: 2 − *x*, −*y*, 1 − *z*)]. Moreover, this complex exhibits π-π stacking interactions involving the plane N(5), C(24)–C(28) of the phen co-ligand in two complex molecules (symmetry code: 1 − *x*, 1 − *y*, 1 − *z*) (angle of interaction of the two planes = 0.0(7)°, centroid-to-centroid distance = 3.603(8) Å, shift distance = 1.218(17) Å) (Figure S6). 

The two copper(II) centres are 4.8067(18) Å apart and display different coordination numbers, one four-coordinate and the other five-coordinate, with the respective coordination geometries being distorted square planar [τ_4_ = {360° − (α + β)}/141° = 0.082, α = 176.3° and β = 172.1°] [78] and distorted square pyramidal [τ_5_ = (β − α)/60° = 0.33, β = 172.4° and α = 152.4°] [74]. The two associated thiosemicarbazonate ligands exhibit different denticities: one coordinates in a tridentate fashion to the metal centre (Cu(2) in Figure 5) with coordination number 4, whereas the other adopts the relatively unusual quadridentate coordination mode to bridge the two metal centres with the thio-enolate nitrogen atom, N(2), and coordinate meridionally to the other metal centre (Cu(1) in Figure 5). For the tridentate ligand, coordinated to Cu(2), the distance of the newly formed thio-enolate N=C bond is virtually indistinguishable from that of the imine C=N bond [*cf.* N(7)–C(38) = 1.316(12) Å vs. C(37)–N(6) = 1.310(12) Å, respectively] and the distance of C(32)–S(2) (1.746(9) Å) lies within the range reported for such thio-enolate bonds. Similarly, for the quadridentate ligand, the distances of the thio-enolate N=C and imine C=N bonds compare favourably [*cf.* N(2)–C(8) = 1.322(13) Å and C(7)–N(1) = 1.301(13) Å, respectively] [66,71,72,73]. The distance of the thio-enolate C–S^−^ bond [C(8)–S(1) = 1.737(9) Å] is normal for complexed thiosemicarbazonate ligands [66,71,72,73]. It is noteworthy that the lengths of the hydrazinic N–N bonds in this dinuclear complex [N(1)–N(2) = 1.381(11) Å and N(6)–N(7) = 1.391(11) Å] are very similar to that observed in the free ligand as a thio-keto (thione) tautomer (1.3854(16) Å), suggesting that there is no delocalisation of electrons involving this chemical bond in the thiosemicarbazonate backbone.

The five-coordinate geometry at Cu(1) is similar to that described for the mononuclear ternary complex (**1**). The bidentate phen co-ligand adopts the axial–equatorial coordination mode. Consequently, the axial Cu–N_phen_ bond is tetragonally elongated, even longer than the Cu–S bond [Cu(1)–S(1) = 1.266(3) Å], causing asymmetric coordination of this co-ligand [Cu(1)–N(4)_eq_ = 2.039(8) Å vs. Cu(1)–N(5) = 2.276(9) Å]. The copper(II) ion, Cu(1), resides 0.177(4) Å above the mean basal plane [N(1), S(1), O(1), N(4)] in the direction of the axial N(5)_phen_ atom. Both copper(II) centres [Cu(1) and Cu(2)], regardless of the differences in the coordination geometries, have a *d_x_*2_–*y*_2 ground state. The literature has witnessed a number of examples of crystallographically characterised dinuclear thiosemicarbazone complexes of copper(II), but these tend to have the same coordination geometry at the two metal centres [8,9,10,72]. To the best of our knowledge, the dinuclear complex [Cu_2_{(3,5-*t*-Bu_2_)-sal4eT}_2_(phen)] (**2**) is one of only two of its kind. The other structurally characterised dinuclear thiosemicarbazone complex of copper(II) featuring two different coordination spheres is [Cu_2_(sal4eT)_2_(bipy)] (Scheme 1), reported as [Cu_2_(L^2^)_2_(bipy)] [4]. Beyond the superficial similarities, there is a sharp distinction between the structures of [Cu_2_{(3,5-*t*-Bu_2_)-sal4eT}_2_(phen)] (**2**) and [Cu_2_(sal4eT)_2_(bipy)] as regards the orientation of the ligands, intermolecular forces and the five-coordinate geometry at one of the two copper(II) centres. Unlike **2**, [Cu_2_(sal4eT)_2_(bipy)] exists as two independent complex molecules, which are similar but not identical, and the thiosemicarbazonate ligands are oriented (differently from **2**) such that *intramolecular hydrogen bonding* occurs between the phenolate oxygen atom bonded to the copper(II) ion in the distorted square planar geometry and the N^4^–H group of the bridging quadridentate ligand. Moreover, the geometries of the five-coordinate copper(II) centres in the two molecules of [Cu_2_(sal4eT)_2_(bipy)] were reported as *distorted trigonal bipyramidal*. We calculated their trigonality indices, τ_5_ [74], to compare them with that of our dinuclear complex (**2**). For [Cu_2_(sal4eT)_2_(bipy)], the values of τ_5_ are ~0.50 and ~0.51 (intermediate between square pyramidal and trigonal bipyramidal); in contrast, for **2**, τ_5_ = 0.33, clearly pointing to greater distortion towards *square pyramidal*. 

### 2.7. X-Band ESR and UV-Visible Spectroscopic Characterisation

**Figure 6:**
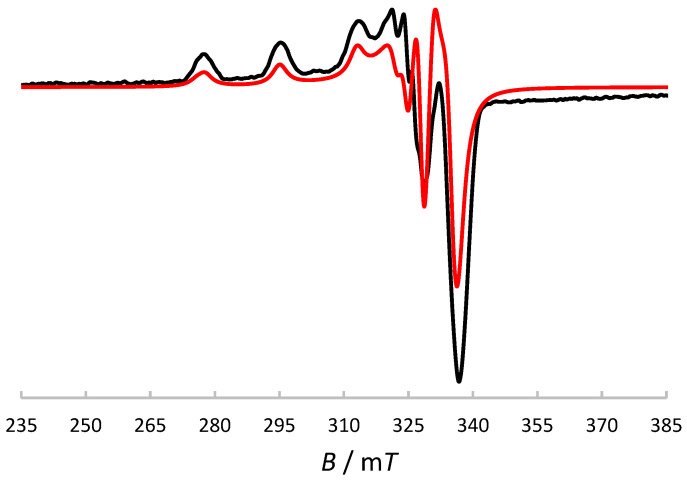
X-band ESR spectrum of [Cu{(3,5-*t-*Bu_2_)-sal4eT}(2,9-Me_2_-phen)] (**1**) recorded in frozen MeOH solution at 77 K (ν = 9.3641 GHz) (black line) and simulation with spin Hamiltonian parameters (red line).

The X-band ESR spectrum of [Cu{(3,5-*t-*Bu_2_)-sal4eT}(2,9-Me_2_-phen)] (**1**) in frozen MeOH solution at 77 K, displayed in Figure 6, is axial. The ESR spin Hamiltonian parameters *g*_ǁ_ = 2.20, *g*_Ʇ_ = 2.05, *A*_ǁ_ = 19.3 mT and *A*_Ʇ_ = 3 mT (*g*_ǁ_ > *g*_Ʇ_ > 2.00; *A*_ǁ_ > *A*_Ʇ_) are consistent with the *d_x_*2_−*y*_2 ground state [75,79,80,81]. Hence, ESR spectroscopy demonstrated that the crystallographically determined distorted square pyramidal geometry at the metal centre is retained in solution. 

**Figure 7:**
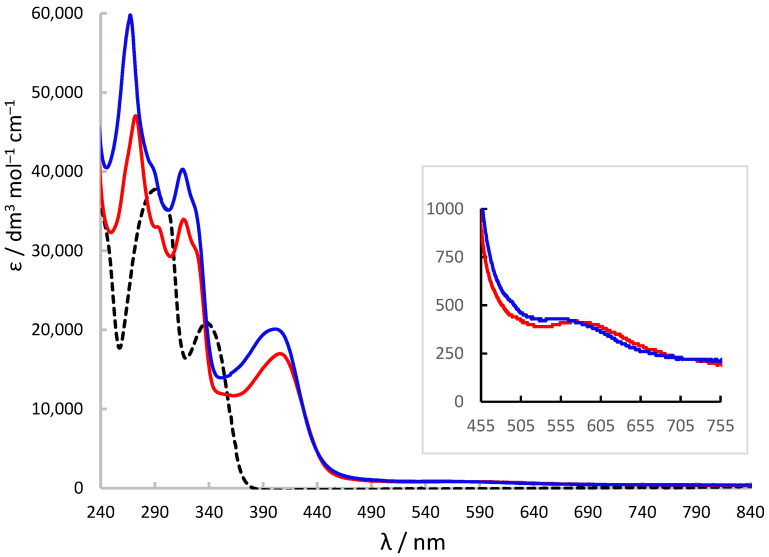
Electronic absorption spectra of H_2_(3,5-*t*-Bu_2_)-sal4eT (dashed line), [Cu{(3,5-*t-*Bu_2_)-sal4eT}(2,9-Me_2_-phen)] (**1**) (red line) and [Cu_2_{(3,5-*t*-Bu_2_)-sal4eT}_2_(phen)] (**2**) (blue line) in MeOH.

Figure 7 shows the electronic absorption spectra of H_2_(3,5-*t*-Bu_2_)-sal4eT, [Cu{(3,5-*t-*Bu_2_)-sal4eT}(2,9-Me_2_-phen)] (**1**) and [Cu_2_{(3,5-*t*-Bu_2_)-sal4eT}_2_(phen)] (**2**). The ligand is colourless; accordingly, its spectrum exhibits UV absorption only. The hugely intense absorption bands are assignable to π → π* (280–305 nm) and *n* → π* (~338 nm) electronic transitions [4]. This pattern of UV absorption is also observed in the spectra of **1** and **2**, albeit at somewhat higher energies.

In the visible region of the electronic spectra of **1** and **2**, there are intense broad bands centred at 405 nm (ε_max_ ~ 17000 dm^3^ mol^−1^ cm^−1^) and 401 nm (ε_max_ ~ 20100 dm^3^ mol^−1^ cm^−1^), respectively, attributable to phenolate/thio-enolate-to-copper(II) charge-transfer electronic transitions [4]. Spin-allowed, but Laporte-forbidden, ligand-field transitions (Figure 7 inset) were observed as weak broad absorption bands at ~590 (ε_max_ ~ 390 dm^3^ mol^−1^ cm^−1^) and ~570 nm (ε_max_ ~ 420 dm^3^ mol^−1^ cm^−1^) for **1** and **2**, respectively. For distorted square-pyramidal copper(II) complexes, such *d*-*d* electronic transitions have been assigned previously as *d_xz_,d_yz_* → *d_x_*2_−*y*_2 in nature [79].

### 2.8. In Vitro Cytotoxicity of the Thiosemicarbazone Ligand and the Ternary Copper(II) Complexes 

**Figure 8:**
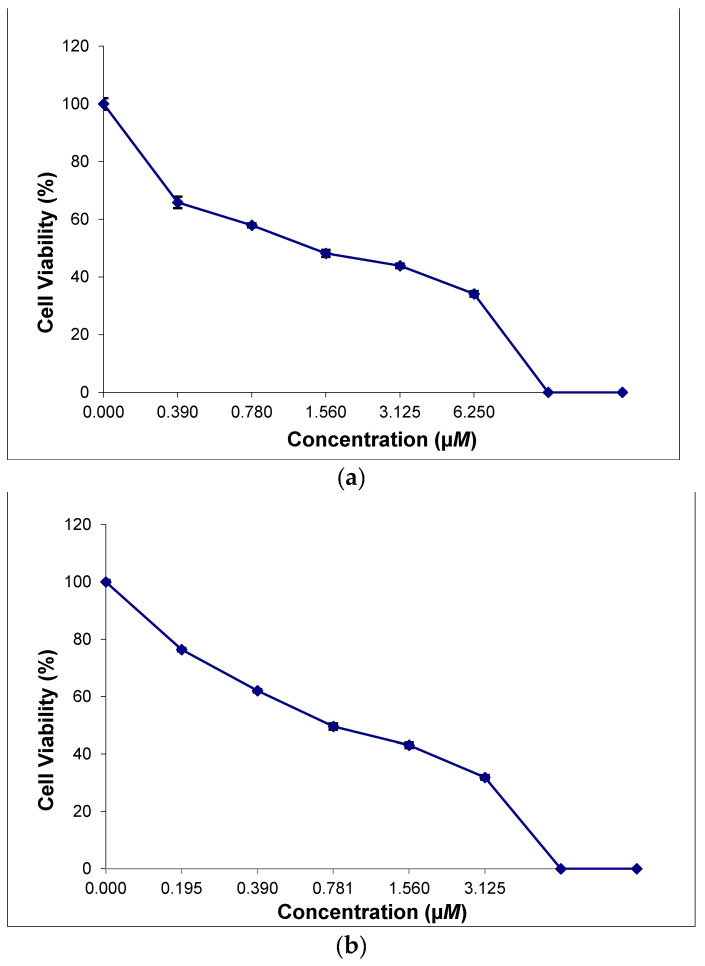
Cytotoxicity of [Cu{(3,5-*t-*Bu_2_)-sal4eT}(2,9-Me_2_-phen)] (**1**) towards HeLa cells (**a**) and [Cu_2_{(3,5-*t*-Bu_2_)-sal4eT}_2_(phen)] (**2**) towards MCF-7 cells (**b**).

The antiproliferative activity of H_2_(3,5-*t*-Bu_2_)-sal4eT, [Cu{(3,5-*t-*Bu_2_)-sal4eT}(2,9-Me_2_-phen)] (**1**) and [Cu_2_{(3,5-*t*-Bu_2_)-sal4eT}_2_(phen)] (**2**) was investigated in two cancer cell lines, namely human cervical carcinoma (HeLa) and human breast adenocarcinoma (MCF-7) using the MTT cell viability assay [MTT = 3-(4,5-dimethylthiazol-2-yl)-2,5-diphenyltetrazolium bromide]. The compounds were dissolved in DMSO (negative control) and it was shown experimentally that the solvent was inactive against the cancer cells (IC_50_ >> 100 μM). The values of the 50% inhibitory concentrations (IC_50_) of these substances together with the positive controls (docetaxel and paclitaxel) were determined, as exemplified for complexes **1** and **2** in the HeLa and MCF-7 cancer cells, respectively (Figure 8). The in vitro antiproliferative potential of each substance was tested within the 0.01–100-μ*M* range of concentrations; the results of these cytotoxicity measurements are presented in Table 4. IC_50_ values for cisplatin were obtained from the literature [82,83]. The details of the MTT cell viability assay are given in the Experiment Section 3.5.

In striking contrast to the marked potent and selective antiproliferative activity of naphthol- and pyridyl-based thiosemicarbazones [11,12], together with their corresponding metal complexes, against tumour cells, the phenolic thiosemicarbazone H_2_(3,5-*t*-Bu_2_)-sal4eT was nontoxic towards both cancer cells in this investigation. However, as is often the case with hydrazones, complexation with metal ions induces pharmacological activity, as can be seen from Table 4. Intriguingly, complex **1** exhibits selective potency towards the Hela cancer cells over the MCF-7 cancer cells. Conversely, the antiproliferative activity of complex **2** is specific towards MCF-7. Although this behaviour has only been observed from tests carried out in vitro, these results show that these copper(II) thiosemicarbazone complexes have potential applications as metallo-drugs in targeted cancer treatment.

Their selectivity over non-cancerous cells such as the human breast epithelial cell line (MCF-10A) has yet to be determined. It is noteworthy that in vitro complexes **1** and **2** are more efficacious as antiproliferative agents than cisplatin. Moreover, cisplatin lacks the cancer-specificity that these complexes possess. They also exhibit higher potent anticancer activity than the standards. Although the study of the mode of action of complexes **1** and **2** as anticancer agents is beyond the scope of this work, it has been amply demonstrated previously for a diverse range of copper(II) complexes, including those of thiosemicarbazones, that the potentials of the Cu^II^/Cu^I^ redox couple [8,15,18,20,30] lie within the biologically accessible redox potential window leading to the generation of reactive oxygen species (ROS) that cause apoptotic cell death.

The differences between the anticancer activities of complexes **1** and **2** are fascinating but complicated. Presumably, they arise from the structural differences (mononuclear vs. dinuclear) and the nature of the bidentate N,N-donor co-ligand (phen vs. 2,9-Me_2_-phen). The cause of cell death is associated with DNA cleavage, ROS generation and the ability of a drug to enter the cancer cell and cause damage. Unfortunately, comparison of this work with previous studies is limited given that [Cu_2_(sal4eT)_2_(bipy)] [4], the only closely related dinuclear complex to [Cu_2_{(3,5-*t*-Bu_2_)-sal4eT}_2_(phen)] (**2**), was not investigated for anticancer activity. Likewise, the reported *crystallographically* characterised ternary *phenolate*-based thiosemicarbazone copper(II) complexes that we are aware of were not tested for cytotoxicity against cancer cells [31,32,33,34,35,36,37,38]. Recently, a paper reported the cytotoxicity of a series of *2-formylpyridyl*-based thiosemicarbazone ternary copper(II) complexes with bipy, phen and their derivatives in the HeLa cell line only and observed the effect of the nature of the co-ligands [39]. It is now well-established, as we also observed in this study, that the antiproliferative activity of thiosemicarbazones is enhanced on complexation with bioactive metal ions. As far as we are aware, *binary* copper(II) complexes of the type [Cu(R^1^,R^2^-bipy/R^1^,R^2^-phen)_n_]^2+^ have not been investigated for anticancer activity. In fact, copper(II) reacts readily with 2,9-Me_2_-phen to form the red copper(I) complex [Cu(2,9-Me_2_-phen)_2_]^+^. We are not aware of any previous studies that compared anticancer activities of *ternary* copper(II) complexes with *binary* copper(II)/bipy or copper(II)/phen complexes. However, it has recently been shown experimentally that free phen, bipy and Cu^II^ exhibit much lower cytotoxicity towards MCF-7 cells than the relevant ternary copper(II) complexes with trien as a primary ligand [53]. Finally, carefully designed series of mononuclear and dinuclear ternary *phenolate*-based thiosemicarbazone complexes similar to **1** and **2**, respectively, are required for a systematic study of antiproliferative activity. Previous studies of such phenolate-containing thiosemicarbazone complexes of copper(II) have focused on DNA cleavage, antimicrobial activity and modelling catalytic activity of metallo-enzymes [33,34,35,36,37].

## 3. Experimental

### 3.1. Materials and Physical Techniques

All pertinent chemicals, reagents and solvents (HPLC/AR-grade) were purchased from Sigma-Aldrich (Burlington, MA, USA) and used as received. Microanalyses (CHN) were performed on a EuroVector elemental analyser (EuroVector, Pavia, Italy). ESI mass spectra were measured with an Agilent 6460 Triple Quadrupole mass spectrometer (Agilent Technologies, Santa Clara, CA, USA) using MeOH as the matrix. Electrical conductivities of the complexes were determined with a JENWAY 4520 conductivity meter (Cole-Parmer, Vernon Hills, IL, USA) at room temperature using freshly prepared solutions (1 mM) in MeOH, EtO, DMF and DMSO. FT-IR spectra were recorded on a Perkin-Elmer spectrophotometer (4000–400 cm^−1^) (Perkin-Elmer, Waltham, MA, USA) with the samples compressed as KBr discs using a Specac press (Specac Ltd., Orpington, UK). ^1^H- and ^13^C-NMR spectroscopic measurements were carried out at room temperature in DMSO-*d*_6_ on a Bruker ASCEN 700 spectrometer (Bruker, Billerica, MA, USA) operating at a radiofrequency of 700 MHz; the chemical shifts are referenced to TMS as an internal standard (δ = 0). X-band ESR spectra of the copper(II) complexes were recorded on a Bruker ELEXSYS E580X FT CW spectrometer (ν ~ 9.4 GHz). Electronic absorption spectra were measured with a Shimadzu 2450 UV-visible spectrophotometer (190–1000 nm) (Shimadzu, Tokyo, Japan) using freshly prepared solutions. Magnetic susceptibility measurements were carried out at room temperature using a Sherwood Scientific magnetic susceptibility balance (Sherwood Scientific, Cambridge, UK). The magnetic data were corrected for diamagnetism using Pascal’s constants in the usual way ($x_{para}=x_{meas}-x_{dia}$). Single-crystal X-ray structural determinations were carried out on a Bruker APEX-II CCD area-detector diffractometer or a Bruker D8 Venture CMOS Photon 100 diffractometer. The crystals were mounted in Fomblin oil and cooled in a stream of cold N_2_. Data were corrected for absorption using empirical methods (SADABS) [84] based upon symmetry equivalent reflections combined with measurements at different azimuthal angles [85]. The crystal structures were solved and refined against *F*^2^ values using ShelXT [86] for solution and ShelXL [87] for refinement (using least squares minimisation), accessed via the Olex2 programme [88]. 

### 3.2. Synthesis of H_2_(3,5-t-Bu_2_)-sal4eT

A sample of 3,5-di-*tert*-butylsalicylaldehyde (2.3433 g, 10.00 mmol) was dissolved in EtOH (30 mL). Separately, 4-ethyl-3-thiosemicarbazide (1.1919 g, 10.00 mmol) was dissolved in EtOH (30 mL). Then, these two solutions were mixed and the resultant solution heated under reflux over a period of three hours. On standing at room temperature for four days, the solution deposited shiny colourless crystals. This product was filtered off on the Büchner funnel, washed with ice-cold EtOH and dried in air (yield: 2.2419 g, 66.82%). Characterisation: calcd for C_18_H_29_N_3_OS (M = 335.50 g/mol): C, 64.44%; H, 8.71%; N, 12.52%. Found: C, 64.17%; H, 8.55%; N, 12.63%; m.p., 218–219 °C. 

### 3.3. Synthesis of [Cu{(3,5-t-Bu_2_)-sal4eT}(2,9-Me_2_-phen)] (**1**) 

To a hot solution of H_2_(3,5-t-Bu_2_)-sal4eT (0.1342 g, 0.40 mmol) in MeOH (30 mL), we added Cu(OAc)_2_·H_2_O (0.0799 g, 0.40 mmol) and 2,9-dimethyl-1,10-phenanthroline (0.0833 g, 0.40 mmol) consecutively with vigorous swirling. The resultant reaction mixture was heated under reflux for 15 min and then filtered and kept at room temperature. Upon slow solvent evaporation from the solution under ambient conditions over a period of one week, shiny black blocks were formed. This crystalline product was isolated through decantation of the mother liquor, washed with ice-cold EtOH and then left to dry in air (yield: 0.2160 g, 89.23%). Characterisation: calcd for C_32_H_39_CuN_5_OS (M = 605.28 g/mol): C, 63.50%; H, 6.49%; N, 11.57%. Found: C, 63.37%; H, 6.50%; N, 11.58%; m.p., 248−249 °C. 

### 3.4. Synthesis of [Cu_2_{(3,5-t-Bu_2_)-sal4eT}_2_(phen)] (**2**)

This complex was synthesised as described for complex **1** above except that 1,10-phenanthroline monohydrate (0.0793 g, 0.4000 mmol) was used as a co-ligand instead of 2,9-dimethyl-1,10-phenanthroline. The resultant dark green solution was heated under reflux for 15 min and then filtered. Large shiny black crystals were obtained from the solution after one week of standing at room temperature. After removal of the supernatant, the crystals were washed with ice-cold EtOH and kept in air (yield: 0.1576 g, 80.86%). Characterisation: calcd for C_48_H_62_Cu_2_N_8_O_2_S_2_ (M = 974.25 g/mol): C, 59.17%; H, 6.41%; N, 11.50%. Found: C, 59.53%; H, 6.29%; N, 11.38%; m.p., 222−224 °C. 

### 3.5. Cell Lines, Cell Culture and Anticancer Activity

The cytotoxicity of the thiosemicarbazone ligand H2(3,5-t-Bu_2_)-sal4eT and its ternary copper(II) complexes [Cu{(3,5-t-Bu_2_)-sal4eT}(2,9-Me_2_-phen)] (**1**) and [Cu_2_{(3,5-t-Bu_2_)-sal4eT}_2_(phen)] (**2**) was tested against two types of cancer cells, namely MCF-7 (human breast adenocarcinoma) and HeLa (human cervical carcinoma), using the MTT cell viability assay [MTT = 3-(4,5-dimethylthiazol-2-yl)-2,5-diphenyltetrazolium bromide], whereby mitochondrial cells in viable cells cleave the tetrazolium rings of the MTT to produce purple membrane-impermeable formazan crystals that readily dissolve in DMSO, and their quantity is determined through visible spectroscopic measurements. The amount of formazan present is a measure of cell viability. The MCF-7 and HeLa cells were cultured in RPMI-1640 medium containing 10% heat-inactivated foetal bovine serum (FBS), penicillin (100 U/mL) and streptomycin (100 μg/mL). 

Three controls were prepared within each 96-well culture plate: DMSO solvent as a negative control and the standards paclitaxel and docetaxel as positive controls. Approximately 10,000 viable cells were seeded per well in 96-well culture and incubated for 24 h at 37 °C and 5% CO_2_. Solutions of the compounds with different concentrations (Figure 8) were prepared in DMSO and added to each well. The cells were then incubated with the compounds for another 48 h, after which the cytotoxicity was evaluated. Initially, the cells were washed with phosphate-buffered saline (PBS) and then 20 μL of the MTT reagent (5 mg/mL) in PBS was added to each well. After 4 h of incubation at 37 °C, the culture medium was discarded, and then the purple formazan crystals were dissolved in DMSO (100 μL) in each well. The absorbance of each well was measured using a plate reader (Anthous 2020; Austria) at λ = 550 nm against a standard reference solution at 690 nm. Assays were carried out in triplicate in three independent experiments. The concentration required for 50% inhibitory activity (IC_50_) was determined from a plot of the percentage cytotoxicity versus the concentration on a logarithmic graph.

## 4. Conclusions

The thiosemicarbazone H_2_(3,5-*t*-Bu_2_)-sal4eT and its ternary copper(II) complexes [Cu{(3,5-*t-*Bu_2_)-sal4eT}(2,9-Me_2_-phen)] (**1**) and [Cu_2_{(3,5-*t*-Bu_2_)-sal4eT}_2_(phen)] (**2**) were synthesised and their chemical identities ascertained through microanalyses, mass spectrometry and vibrational spectroscopy. Definitive evidence for their 3D structures was obtained through single-crystal X-ray analyses. As is commonly the case with phenolic thiosemicarbazones, this ligand was isolated as the thio-keto (thione) tautomer and in the *E*-configuration with respect to the Schiff-base imine bond. ^1^H-NMR spectroscopy showed that this isomeric form is maintained in solution. The X-ray structures of complexes [Cu{(3,5-*t-*Bu_2_)-sal4eT}(2,9-Me_2_-phen)] (**1**) and [Cu_2_{(3,5-*t*-Bu_2_)-sal4eT}_2_(phen)] (**2**) showed that the thiosemicarbazone underwent base-/metal-assisted tautomerisation upon coordination to copper(II); moreover, this ligand also demonstrated coordination versatility in that, in complex **2**, it employed two different denticities, namely tridentate and quadridentate. The planarity and rigidity of the thiosemicarbazonate ligand and the co-ligand 2,9-dimethyl-1,10-phenanthroline imposed a distorted square-pyramidal geometry at the metal centre of complex **1** (τ = 0.14), whereby the donor atoms of the tridentate primary ligand are arranged meridionally, whereas the bidentate co-ligand adopts an axial–equatorial coordination mode. As always observed in X-ray structures of this type of ternary copper(II) complexes, there is considerable elongation of the axial coordinate bond in conformity with the tetragonal distortion. The ESR spectrum of **1** in frozen solution is axial (*g*_ǁ_ > *g*_Ʇ_ > 2.00; *A*_ǁ_ > *A*_Ʇ_) and indicative of half occupancy of the *d_x_*2_−*y*_2 orbital. The dinuclear complex (**2**) exhibits different coordination geometries at the copper(II) centres, namely distorted square planar and distorted square pyramidal. Such dinuclear ternary copper(II) complexes are few and far between. Intriguingly, the cytotoxic activities of [Cu{(3,5-*t-*Bu_2_)-sal4eT}(2,9-Me_2_-phen)] and [Cu_2_{(3,5-*t*-Bu_2_)-sal4eT}_2_(phen)] in the HeLa and MCF-7 cancer cell lines are vastly different. Whereas the former is highly antiproliferative against HeLa cancer cells but non-toxic towards MCF-7 cancer cells, the converse is true for the latter. Such selective cytotoxicity of **1** and **2** towards these cancer cells is not shown with cisplatin. Considering that the primary ligand is the same in complexes **1** and **2**, the difference in the pharmacological behaviour probably derives from structural differences and the nature of the co-ligand. A series of complexes of this type are required to draw appropriate conclusions.

## Figures and Tables

**Table 1:** Selected crystallographic data for H_2_(3,5-*t*-Bu_2_)-sal4eT and complexes **1** and **2**.

Compound	H_2_(3,5-*t*-Bu_2_)-sal4eT	1	2
Chemical formula	C_18_H_29_N_3_OS	C_32_H_39_CuN_5_OS	C_48_H_62_Cu_2_N_8_O_2_S_2_
Molar mass (g mol^−1^)	335.50	605.28	974.25
*T* (K)	100	99.99	100.01
Crystal system	monoclinic	monoclinic	triclinic
Space group	*P*2_1_/*c*	*P*2_1_/*n*	*P* $\bar{1}$
*a* (Å)	18.4754(14)	8.5924(3)	11.3898(6)
*b* (Å)	9.2040(7)	18.5809(6)	13.1195(8)
*c* (Å)	11.5714(9)	19.4132(6)	16.6701(10)
*α* (°)	90	90	78.980(2)
*β* (°)	95.517(2)	100.451(2)	89.824(2)
*γ* (°)	90	90	83.661(3)
*V* (Å^3^)	1958.6(3)	3047.99(17)	2429.7(2)
*Z*	4	4	2
*ρ*_calc_ (g cm^−3^)	1.138	1.319	1.332
*μ* (mm^−1^)	0.173	1.904	2.241
*F*(000)	728.0	1276.0	1024.0
Crystal size (mm)	0.500 × 0.200 × 0.150	0.240 × 0.120 × 0.080	0.508 × 0.207 × 0.040
Radiation (λ/Å)	MoK*α* (*λ* = 0.71073)	CuK*α* (*λ* = 1.54178)	CuK*α* (*λ* = 1.54178)
2*Θ* range (°)	4.43–57.282	6.638–133.168	5.402–133.766
Reflections collected	37828	19602	30501
*R*_int_	0.0661	0.0918	0.0708
GOF on *F*^2^	1.051	1.044	1.189
*R*_1_, *wR*_2_ (*I* ≥ 2*σ* (*I*))	0.0403, 0.0909	0.0513, 0.1235	0.1083, 0.3568
*R*_1_, *wR*_2_ (all data)	0.0607, 0.1010	0.0749, 0.1378	0.1180, 0.3692

**Table 2:** Selected bond distances (Å) and angles (°) for H_2_(3,5-*t*-Bu_2_)-sal4eT.

C(16)–S(1)	1.7029(14)
C(1)–O(1)	1.3605(16)
N(1)–N(2)	1.3854(16)
C(15)–N(1)	1.2904(19)
C(16)–N(2)	1.3499(18)
C(15)–N(1)–N(2)	113.66(12)
N(2)–C(16)–N(3)	118.40(13)
N(2)–C(16)–S(1)	117.87(11)
N(3)–C(16)–S(1)	123.72(11)

**Table 3:** Selected bond distances (Å) and angles (°) for **1** and **2**.

[Cu{(3,5-*t*-Bu_2_)-sal4eT}(2,9-Me_2_-1,10-phen)] (1)			
Cu(1)–S(1)	2.2823(9)	S(1)–C(8)	1.739(3)
Cu(1)–O(1)	1.934(2)	N(2)–C(8)	1.313(4)
Cu(1)–N(1)	1.959(3)	N(1)–N(2)	1.400(4)
Cu(1)–N(4)	2.057(3)	N(1)–C(7)	1.296(4)
Cu(1)–N(5)	2.308(3)	N(3)–C(8)	1.357(4)
O(1)–Cu(1)–N(1)	91.55(10)	N(1)–Cu(1)–N(4)	172.64(11)
N(1)–Cu(1)–S(1)	84.77(8)	N(1)–Cu(1)–N(5)	109.81(11)
O(1)–Cu(1)–N(4)	90.08(10)	N(4)–Cu(1)–S(1)	91.76(7)
O(1)–Cu(1)–N(5)	98.14(10)	N(5)–Cu(1)–S(1)	97.38(7)
O(1)–Cu(1)–S(1)	164.38(8)	N(4)–Cu(1)–N(5)	77.04(11)
**[Cu_2_{(3,5-*t*-Bu_2_)-sal4eT}_2_(phen)] (2)**			
Cu(1)–S(1)	2.266(3)	Cu(2)–S(2)	2.231(3)
Cu(1)–O(1)	1.908(7)	Cu(2)–O(2)	1.888(7)
Cu(1)–N(1)	1.952(8)	Cu(2)–N(2)	2.017(8)
Cu(1)–N(4)	2.039(8)	Cu(2)–N(6)	1.940(8)
Cu(1)–N(5)	2.276(9)	N(6)–C(37)	1.310(12)
S(1)–C(8)	1.737(9)	S(2)–C(38)	1.746(9)
N(2)–C(8)	1.322(13)	N(7)–C(38)	1.316(12)
N(1)–C(7)	1.301(13)	N(6)–N(7)	1.391(11)
N(1)–N(2)	1.381(11)	N(8)–C(38)	1.353(13)
O(1)–Cu(1)–N(1)	93.7(3)	N(5)–Cu(1)–S(1)	102.9(2)
N(1)–Cu(1)–S(1)	85.2(2)	N(4)–Cu(1)–N(5)	77.7(3)
O(1)–Cu(1)–N(4)	89.7(3)	O(2)–Cu(2)–N(2)	88.1(3)
O(1)–Cu(1)–N(5)	104.6(3)	O(2)–Cu(2)–N(6)	94.2(3)
O(1)–Cu(1)–S(1)	152.4(2)	N(2)–Cu(2)–N(6)	176.3(3)
N(1)–Cu(1)–N(4)	172.2(4)	S(2)–Cu(2)–N(2)	91.8(2)
N(1)–Cu(1)–N(5)	94.6(3)	S(2)–Cu(2)–N(6)	86.3(2)
N(4)–Cu(1)–S(1)	95.1(2)	S(2)–Cu(2)–O(2)	172.1(3)

**Table 4:** Cytotoxicity evaluation of H(3,5*-t*-Bu_2_)-sal4eT, **1** and **2** via MTT assay in cancer cell lines over a 24 h incubation period.

	IC_50_/µM	
Compound	HeLa	MCF-7
H(3,5*-t*-Bu_2_)-sal4eT	>100	>100
[Cu{3,5-*t*-Bu_2_)-sal4eT}(2,9-Me_2_-phen)] (**1**)	1.35 ± 0.11	>100
[Cu_2_{3,5-*t*-Bu_2_)-sal4eT}_2_(phen)] (**2**)	>100	0.73 ± 0.06
Docetaxel	60.70 ± 5.13	92.54 ± 7.99
Paclitaxel	15.84 ± 1.28	>100
Cisplatin	13.28 ± 3.84 [82]	13.36 ± 1.25 [83]

## Data Availability

Data are contained within the article.

## Supplementary Materials

The following supporting information can be downloaded at: https://www.mdpi.com/article/10.3390/molecules29020431/s1, CCDC 2323038–2323040 contain the supplementary crystallographic data for H_2_(3,5-*t*-Bu_2_)-sal4eT, **1** and **2**, respectively, in this paper. These data can be obtained free of charge at http://www.ccdc.cam.ac.uk/data_request/cif, by emailing data_request@ccdc.cam.ac.uk or by contacting the Cambridge Crystallographic Data Centre, 12 Union Road, Cambridge CB2 1EZ, UK; fax: +44 1223 336033. Figures S1–S6 are available as Supplementary Information.
