# Supplementary material for: Ternary Phenolate-Based Thiosemicarbazone Complexes of Copper(II): Magnetostructural Properties, Spectroscopic Features and Marked Selective Antiproliferative Activity against Cancer Cells
Source: Molecules. 2024 Jan 16;29(2):431. doi: 10.3390/molecules29020431 (PMC10819714; doi:10.3390/molecules29020431)
Supplement: Supplementary file 1 [file molecules-29-00431-s001.zip › molecules-2808450-supplementary.pdf]

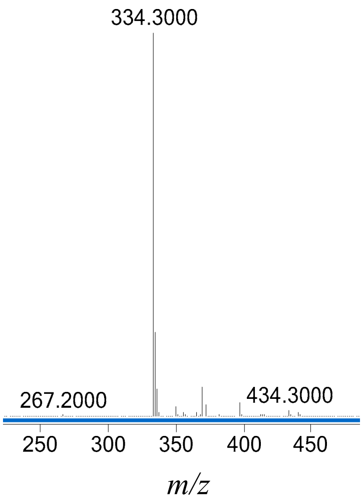

Figure S1. ESI mass spectrum of H_2(3,5-*t*-Bu_2)-sal4eT in the negative mode.

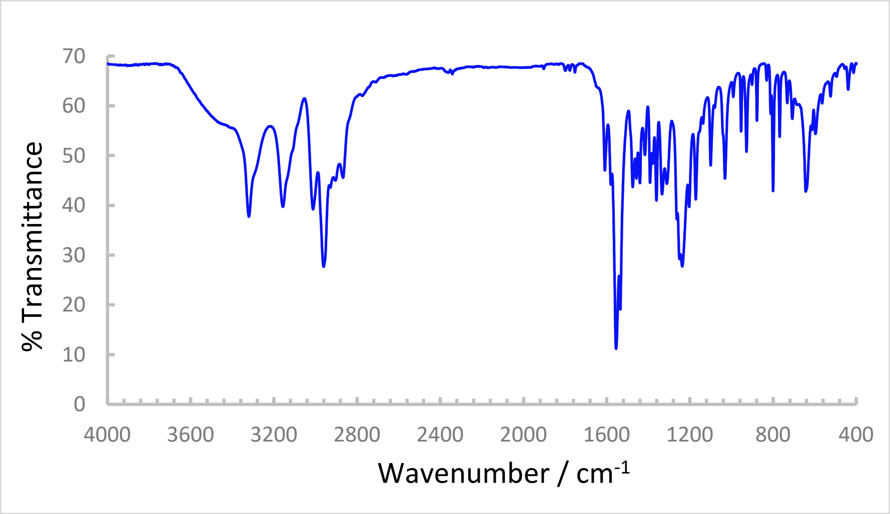

(a)

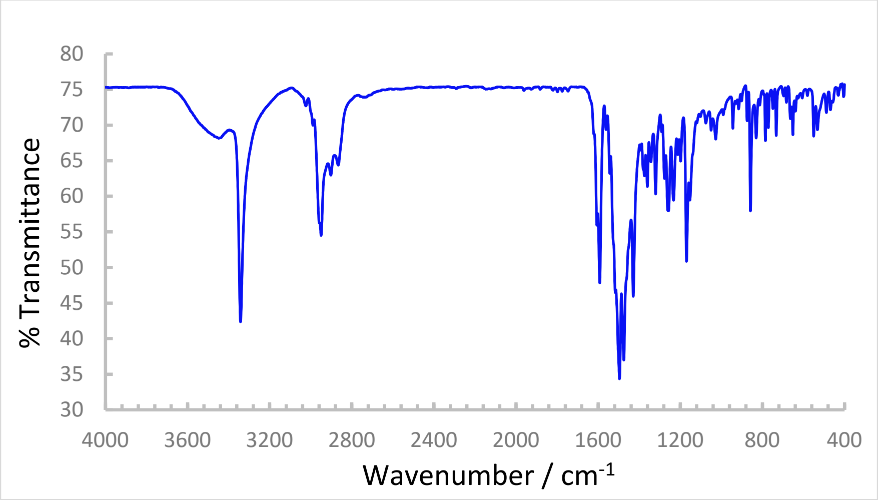

(b)

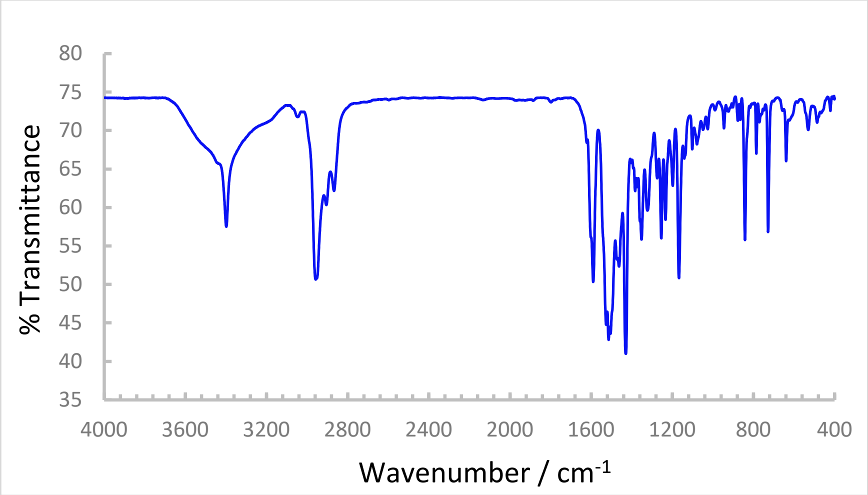

(c)

Figure S2. FT-IR spectra of (a) H_2(3,5-*t*-Bu_2)-sal4eT, (b) complex **1** and (c) complex **2.**

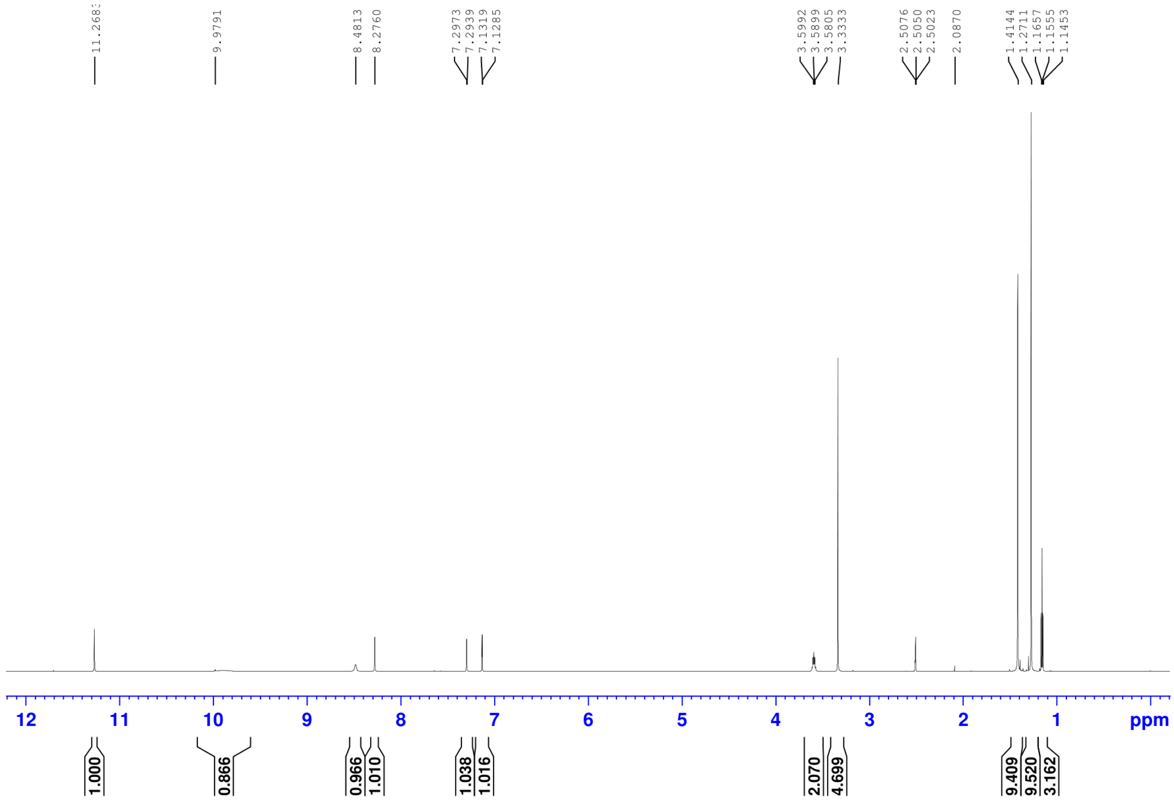

(a)

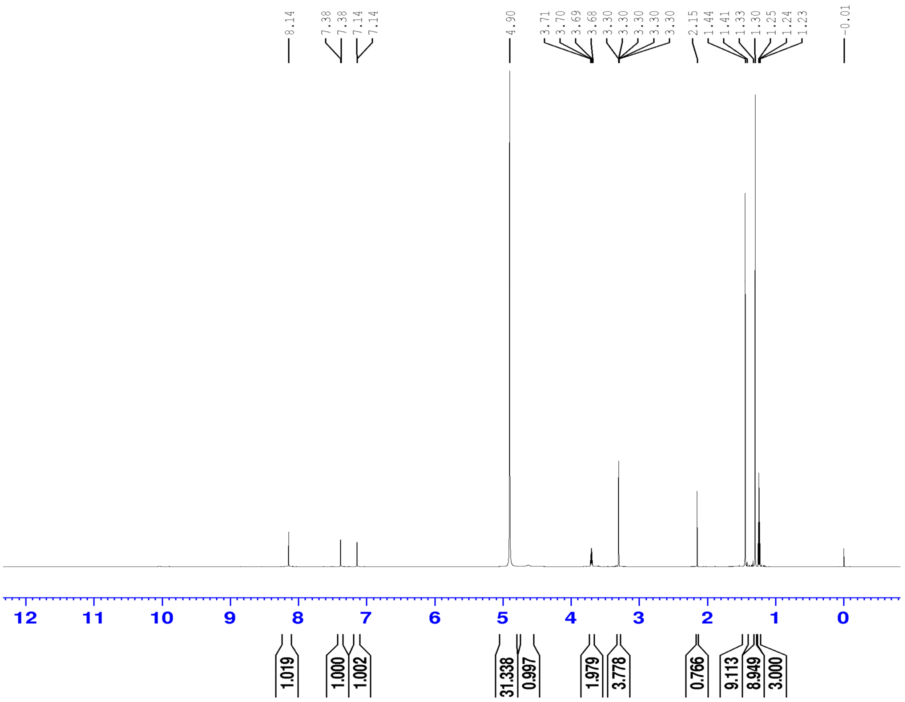

(b)

Figure S3. ^{1}H-NMR spectra of H_2(3,5-*t*-Bu_2)-sal4eT in (a) DMSO-d_6 and (b) CD_3OD (700 MHz)

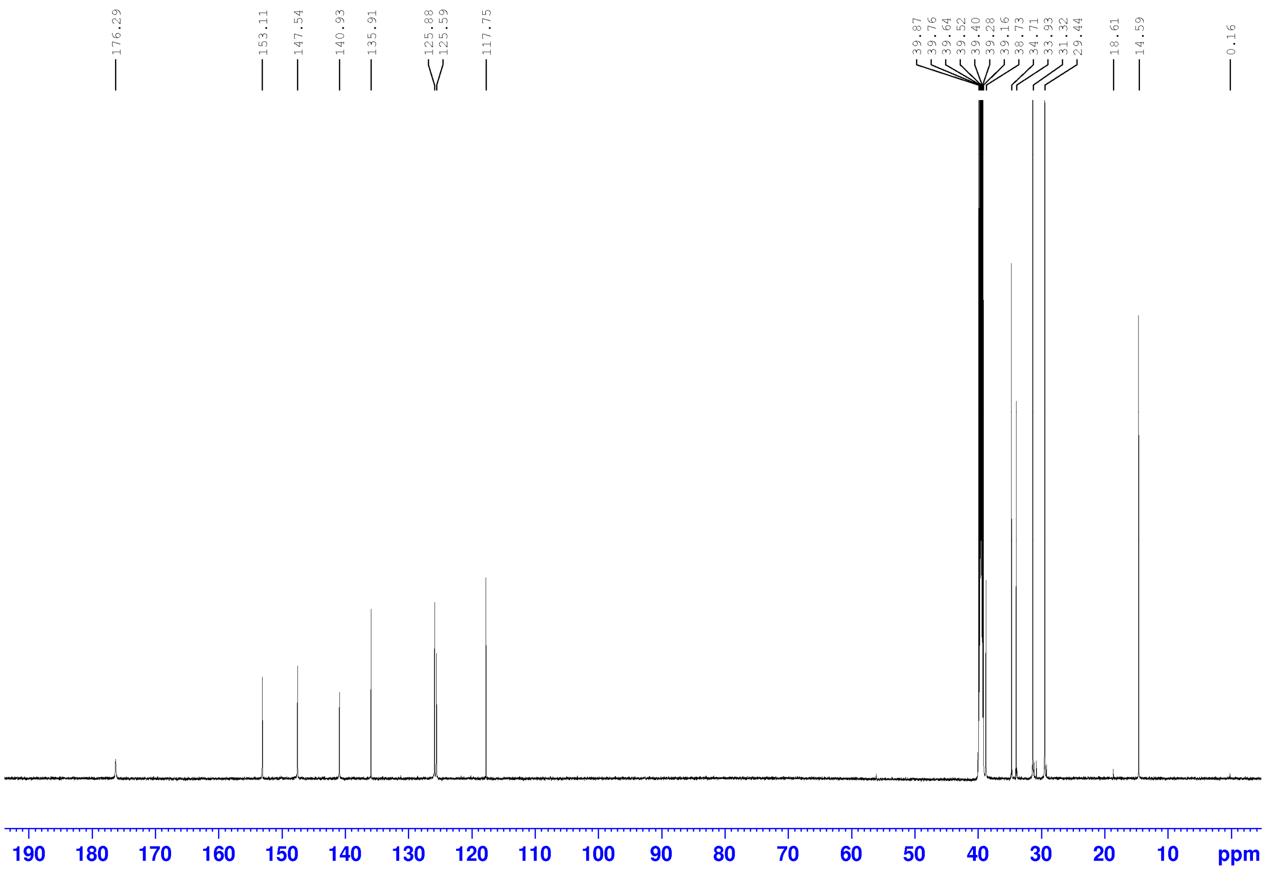

Figure S4. ^{13}C-NMR spectrum of H_2(3,5-*t*-Bu_2)-sal4eT in DMSO-d_6 (176 MHz)

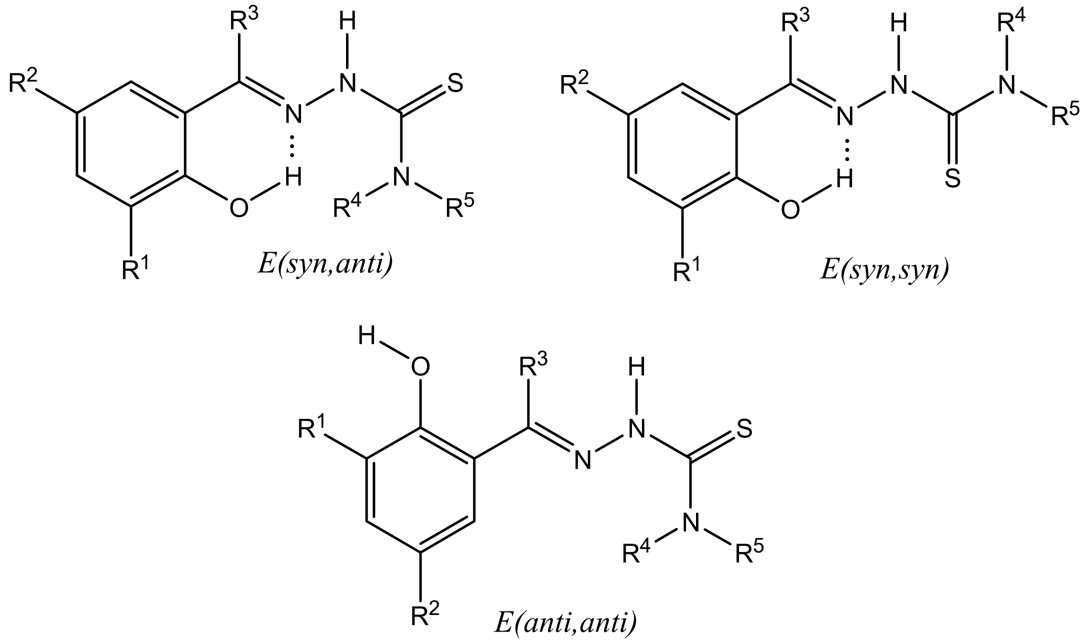

Figure S5. Representations of crystallographically observed different orientations of phenolic thiosemicarbazones.

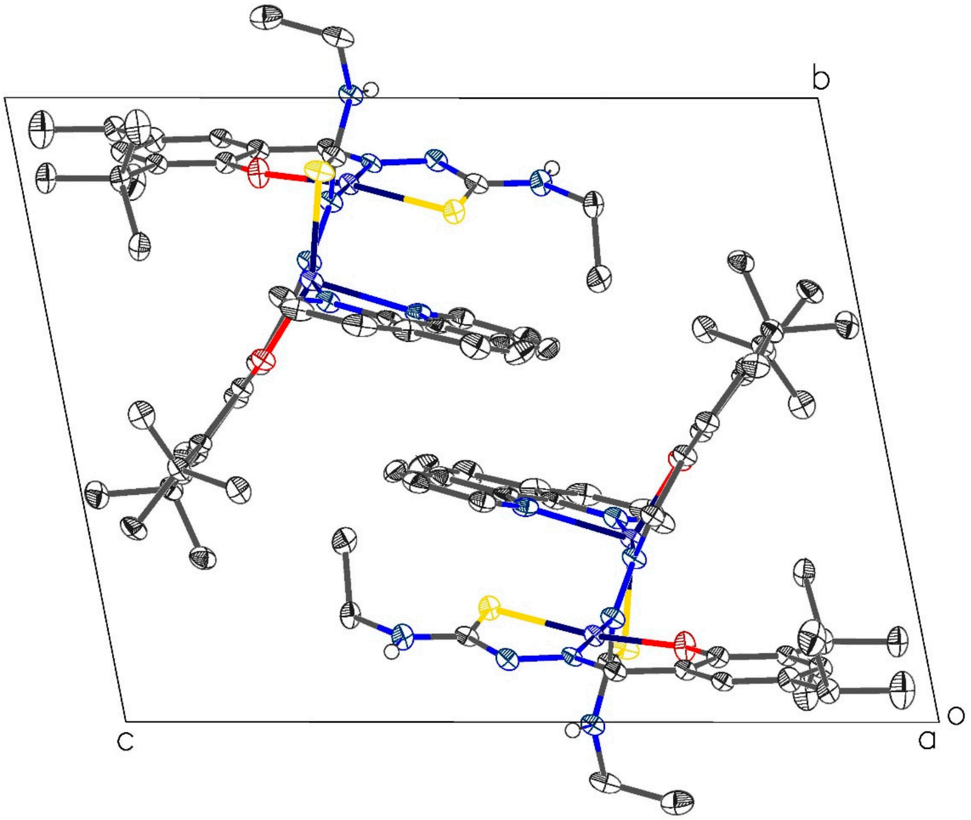

Figure S6. Unit cell π-π stacking interaction in [Cu_2{(3,5-*t*-Bu_2)-sal4eT}$_2$(phen)] (**2**).
